# Hydrogen sulfide attenuates isoflurane-induced neuroapoptosis and cognitive impairment in the developing rat brain

**DOI:** 10.1186/s12871-017-0419-y

**Published:** 2017-09-05

**Authors:** Xueyuan Hu, Li Luan, Wei Guan, Shuai Zhang, Bei Li, Wei Ji, Honggang Fan

**Affiliations:** Department of Veterinary Medicine, College of Veterinary Medicine, Northeast Agricultural University, No. 600 of Changjiang Road, Xiangfang District, Harbin, Heilongjiang Province China

**Keywords:** Hydrogen sulfide, Isoflurane, Neuroapoptosis, Cognitive impairment

## Abstract

**Background:**

Isoflurane-induced neuroapoptosis and cognitive impairment has been previously reported. Hydrogen sulfide (H_2_S) has been shown to be a neuromodulator that is thought to have anti-apoptotic, anti-inflammatory, and anti-oxidative benefits. However, it is not known if H_2_S is protective against anesthesia-induced apoptosis and cognitive defects.

**Methods:**

In this study, postnatal day 7 (P7) Sprague-Dawley rats were randomly divided into four groups: control group (normal saline), H_2_S group (NaHS 28 μM/kg), isoflurane group (normal saline +0.75% isoflurane) and H_2_S preconditioning group (NaHS 28 μM/kg + 0.75% isoflurane). After exposure to isoflurane for 6 h, half the numbers of rats in each group were euthanized, and the hippocampus and cerebral cortex were dissected and examined for apoptosis by the terminal deoxynucleotidyl transferase-mediated dUTP nick end-labeling (TUNEL) technique and western blot. After 6 weeks, the remaining rats were subjected to a Morris water maze (MWM) test for behavioral assessment.

**Results:**

The TUNEL assay and western blot showed that when rats were preconditioned with NaHS, neuroapoptosis decreased significantly both in hippocampus and cerebral cortex compering with the isofulrane group. The MWM showed that P7 rats administration of NaHS improved cognitive impairments induced by isoflurane.

**Conclusions:**

The current study demonstrates that H_2_S attenuates isoflurane-induced neuroapoptosis and improves cognitive impairments in the developing rat brain.

## Background

Isoflurane and other inhaled anesthetics are widely used on children for various types of surgeries. Although some studies have shown neuroprotection by anesthesia in in vitro and in vivo hypoxia/ischemia models [1, 2], numerous reports and some retrospective clinical studies have shown that inhaled anesthetics can induce apoptosis and can be detrimental to cognitive development [3–6], especially during the “brain growth spurt” stage, which occurs from P2 to P14 after birth in rodents [7]. Isoflurane modulates specific ligand-gated ion channels. It is principally an agonist for the γ-aminobutyric acid (GABA) type A receptor [8], thereby altering synaptic function [9]. Synaptic changes can result in long-term consequences for the developing brain, as was first identified with the deleterious effects of alcohol during neurodevelopment [10]. It has been reported that immature rats exposed to isoflurane have persistent memory and learning impairments, which are associated with widespread caspase-3 activation and neuronal apoptosis [3, 6, 11].

Hydrogen sulfide (H_2_S) is involved in many biological systems, and like nitric oxide and carbon monoxide, it is a signaling molecule in mammalian cells [12]. H_2_S is widely recognized as a neuromodulator, and has been found at high levels in the hippocampus and cerebellum [13]. Exogenous H_2_S donors have been used in many animal models to mimic the physiological and biological effects of endogenous H_2_S, which ranges in concentrations from 0.1 μM to more than 300 μM [14], although high concentrations of H_2_S can detrimentally affect cytochrome oxidase in many tissues [15]. In the central nervous system, endogenous H_2_S potentiates N-methyl-D-aspartate receptor-mediated currents and long-term potentiation (LTP), enhances the effect of GABA, regulates intracellular calcium levels, and stimulates uptake of synaptic glutamate by astrocytes [16, 17]. In addition, H_2_S-releasing drugs have been shown to affect a wide range of physiological responses, such as blood vessel relaxation [18], neurotransmission [13], regulation of inflammation [19], cardioprotection [20], and insulin release [21]. In light of their many physiological effects, several H_2_S drugs are in clinical trials [22].

In recent years, researchers have demonstrated that xenon [23], dexmedetomidine [24] and isoflurane preconditioning [25] attenuate anesthesia-induced apoptosis and cognitive dysfunction in the developing rat brain. However, it has not been reported if H_2_S can reduce anesthesia-induced apoptosis and cognitive defects. In this study we investigated if NaHS (a H_2_S donor) could reduce neuronal injury and improve learning and memory caused by exposure to isoflurane at an early age. We feel that this study may offer new insights on treatments to help protect against neuroapoptosis and improve cognitive dysfunction caused by exposure to anesthetics.

## Methods

### Animals

Postnatal day 7 (P7) Sprague-Dawley rat pups of both sexes, weighing 16–18 g, provided by Harbin Medical University Experimental Animal Center. All experimental protocols were approved by the Animal Care and Use of Northeast Agricultural University Committee. Pups were housed in polypropylene cages, and allowed to reunite with their mothers after anesthesia. Rat were housed with 45%–55% humidity in 12-h light-dark cycle. Food and water were available freely and the room temperature was maintained at 22 °C–24 °C.

### Anesthesia and treatment

All rats were randomly assigned into four groups (*n* = 12): a control group (Control [CON]), a H_2_S group (NaHS 28 μM/kg [H_2_S]), an isoflurane group (0.75% isoflurane [ISO]) and a H_2_S-treated group (NaHS 28 μM/kg + 0.75% isoflurane [H_2_S + ISO]). Before (30 min prior) exposure to 0.75% isoflurane (Yipin Co., Ltd., Hebei, China), a single bolus of normal saline was injected intraperitoneally in rats in the CON and ISO groups. NaHS at 28 μM/kg doses was (Sigma-Aldrich, St Louis, MO, USA) injected intraperitoneally in the H_2_S and H_2_S + ISO groups, respectively. Isoflurane was administered in an anesthesia chamber with 0.75% anesthetic mixed with 100% oxygen using an agent-specific vaporizer for ISO and H_2_S+ISO groups. Anesthesia was administered using a customized closed-circuit system, and carbon dioxide was eliminated with soda lime.

After 6 h exposure, half of the rat pups from each group were euthanized by intraperitoneal injection of pentobarbital sodium (150 mg/kg; Sigma, St. Louis, MO) overdose and the brains were dissected following an anatomical atlas as previously described [26]. Following decapitation, the left cerebral cortex and hippocampus of each brain were immediately frozen in liquid nitrogen and stored at −80 °C for protein analysis. The contralateral side was post-fixed in 4% paraformaldehyde saline and kept at 4 °C for neuronal apoptosis analysis. The remaining animals were used for a behavioral study to determine their cognitive function after 6 weeks.

### TUNEL staining analyses

After overnight fixation in 4% paraformaldehyde solution, the samples were embedded in paraffin blocks (5 μm). Wax-embedded tissue sections were dewaxed and the brain sections were subjected to terminal deoxynucleotidyl transferase (TdT)-mediated dUTP nick end-labeling (TUNEL) using an Apop Tag plus peroxidase in situ kit (Keygen Biotechnology Co., Ltd., Nanjing, China). TUNEL staining shows DNA fragmentation and is recognized as a standard technique for the detection of apoptosis in tissue sections [27]. Following the manufacturer’s instructions, paraffin blocks were immersed in 1% Triton X-100 (5 min, room temperature [RT]), 3% H_2_O_2_ (10 min, RT), 100 μl proteinase K (30 min, 37 °C), and 100 μl DNase I reaction solution (30 min, 37 °C), then 50 μl TdT enzyme reaction solution (containing biotin-labeled dUTP, 60 min, 37 °C) and 50 μl horseradish peroxidase-conjugated streptavidin (37 °C) was added in sequence, and incubated in the dark. Tissue sections were treated with 3, 3′-diaminobenzidine chromogen for approximately 5 min then counterstained with hematoxylin. For the control groups, sections were stained using the same procedures in the absence of DNase I and TdT reaction solutions. The numbers of TUNEL-positive cells were examined by an observer who was blind to the group assignment of the sections. Three sections were selected randomly from each sample and the numbers of positive cells (neurons) in the cerebral cortex and hippocampus were counted for each section at a high-magnification field (×400) in five visual fields. The formula used for assessing apoptosis was apoptotic index = the number of TUNEL-positive nuclei × 100% / the total number of nuclei.

### Western blot analyses

Brain tissues were harvested and homogenized on ice before centrifugation at 13,000×g at 4 °C for 5 min. The supernatants were collected and saved at −20 °C. Total protein concentration was measured with the bicinchoninic acid method (Beyotime Biotechnology, Co., Ltd., Jiangsu, China). The samples were separated using 12% gel electrophoresis (90 V for 25 min, then 120 V for 1 h) and then transferred to a nitrocellulose membrane at 200 mA for 1 h. The blots were blocked in 5% milk Tris-buffered saline and Tween-20 (TBST) buffer for 1.5 h at 25 °C, and then incubated with a monoclonal antibody against caspase-3 (1:1000; Cell Signaling Technology, Inc. #9662, Danvers, MA, USA) or β-actin (1:1000; Cell Signaling Technology) in 5% milk TBST buffer at 4 °C overnight. After washing in TBST, membranes were incubated with horseradish peroxidase-conjugated goat anti-rabbit IgG antibody (1:6000 dilution for β-actin and caspase-3, Wanleibio Co., Ltd., Shenyang, China) for 2 h at RT. Following the ECL reaction, the membranes were subjected to autoradiography and films were scanned using Tanon Imager 5200 software v2.03 (Tanon Co., Ltd., Shanghai, China). Western blot band intensity was quantified by the mean pixel intensity using Gel-Pro Analyzer 4 software (Media Cybernetics Inc., Bethesda, MD, USA).

### Learning and memory tests

The Morris water maze (MWM) test has been described in multiple reviews to assess neural basis of allocentric learning and memory [28]. Briefly, as described previously [29], a round pool, 150 cm diameter and 50 cm depth, separated into quadrants, was filled with opaque water (24 °C) to a height of 1.5 cm above the top of a movable clear 15-cm-diameter platform in a designated third quadrant. The pool was surrounded by a dark black curtain to prevent confounding visual cues. A video tracking system recorded the time it took the rats to reach the platform, and the data were analyzed using motion detection software for the MWM (Xinxin Software, Shanghai, China). The P42 rats were trained for 5 days to determine the ability of the rats to obtain spatial information. All rats were subjected to two trials per day in each of the four quadrants of the swimming pool. For each trial, rats were placed at a fixed position in the swimming pool, facing the wall, and allowed to search for the platform in the third quadrant for up to 90 s. If a rat did not find the platform within 90 s, the rat was gently guided to the platform and allowed to remain there for 20 s. For all training trials, swimming speed and the time to reach the platform (escape latency time) were recorded. If a rat took less time to reach the platform, the learning ability was considered to be improved. We took the average time of two trials as the escape latency time in each day. After the 5-day training, probe trials were assessed immediately to evaluate memory retention capability. The probe trials involved removing the submerged platform in the third quadrant from the pool and allowing the rats to swim for 90 s in any of the four quadrants of the pool. The number of times the rats crossed the original platform position and the time they spent in the object quadrant were recorded. After every trial, each rat was wiped dry and kept warm before returning it to its cage.

### Statistical analysis

GraphPad Prism 7 (GraphPad Software for Windows Inc., San Diego, CA, USA) was used for statistical analysis and graph generation. The average number of platform crossings were expressed as median and interquartile range and analyzed using the Mann–Whitney U test. The escape latency and other assessments were expressed as mean ± standard deviation (SD) and determined by two-way repeated factor analysis of variance (ANOVA) with Student-Newman-Keuls or Bonferroni’s tests. In all experiments, differences were considered statistically significant at *p* < 0.05 and extremely significant at *p* < 0.01.

## Results

### H_2_S mitigates neuronal apoptosis induced by 0.75% isoflurane

**Fig. 1:**
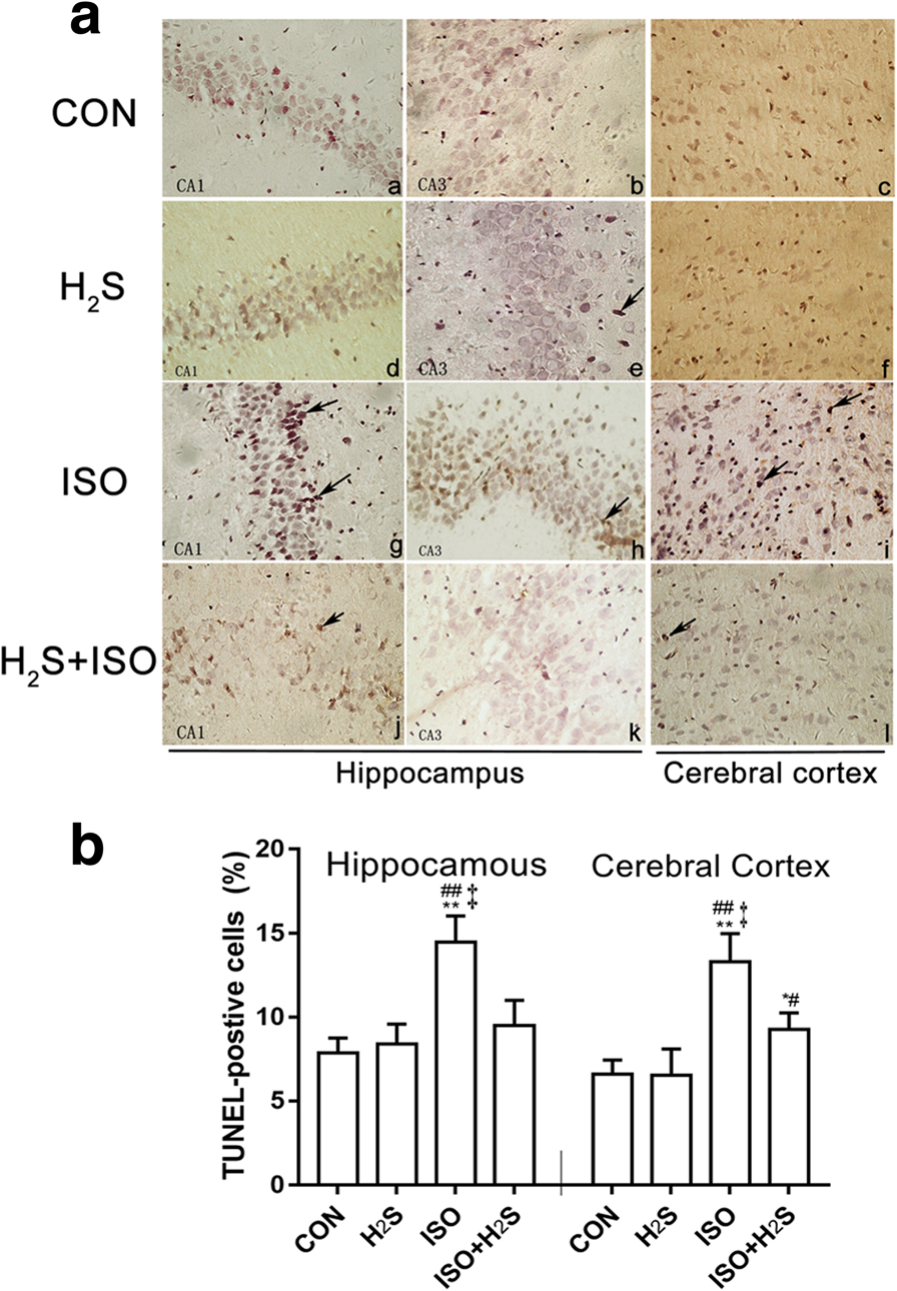
H2S attenuates isoflurane-induced neuro-apoptosis in hippocampus and cerebral cortex tissue. Paraffin sections were stained with the TUNEL technique using Apop Tag kit and counterstained with hematoxylin (**A**), under a high magnification (400×). For positive cells, nuclei stained dark brown with irregular or disintegration of different apoptotic bodies (indicated by the arrows); as for negative cells with nuclei light blue and regular. Quantification of apoptosis following TUNEL staining (**B**), calculating the apoptotic indices, expressed as Mean ± SD of five 400× fields in each rat (*n* = 6). * *p* < 0.05, ** *p* < 0.01 presented a significant difference compared with CON group; # *p* < 0.05, ## *p* < 0.01 presented a significant difference compared with H_2_S group, ‡ *p* < 0.01 presented a significant difference between ISO group and ISO + H_2_S group

No signs of abdominal pain or discomfort were observed after administration of NaHS or normal saline, and none of the rats died. After exposure to 0.75% isoflurane for 6 h, TUNEL-positive cells were quantitated in all assessed brain samples (Fig. 1A), and compared with the CON group, neuronal apoptosis was higher (Fig. 1A: d, e, f) in both the hippocampus and cerebral cortex. However, the H_2_S-treated group showed neuro-protection, compared with the ISO group, with less nuclei shrinkage or broken or destroyed nuclei, especially in the hippocampal CA3 region and cerebral cortex (Fig. 1A: g, h, i, j, k, l). Quantification of apoptotic index (Fig. 1B) revealed that the number of TUNEL-positive cells increased significantly in the ISO group, compared with the CON group, in the cerebral cortex (*p* < 0.01) and in the hippocampus (*p* < 0.01). However, the number of TUNEL-positive cells in the H_2_S treatment group was reduced significantly (cortex, *p* < 0.01; hippocampus, *p* < 0.01, vs the ISO group), although it was still greater than the CON group in both the cortex (*p* < 0.05). There was no significant differences between the H_2_S group and CON group.

**Fig. 2:**
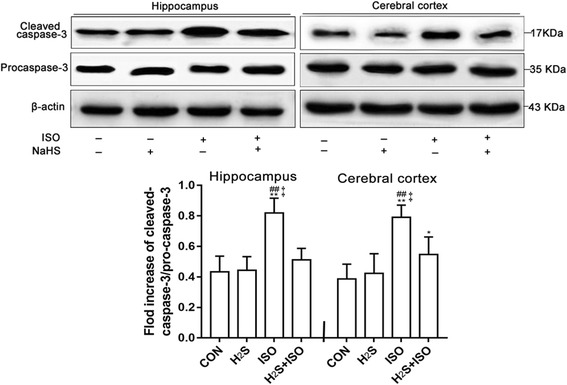
H2S attenuates isoflurane-induced cleaved caspase-3 in hippocampus and cerebral cortex at an early age. Results are presented as the ratio of the intensity of the activation caspase-3 band to the intensity of the pro-caspase-3 band. Data are presented as the Mean ± SD (*n* = 6). * *p* < 0.05, ** *p* < 0.01 presented a significant difference compared with CON group; ## *p* < 0.01 presented a significant difference compared with H_2_S group, ‡ *p* < 0.01 presented a significant difference between ISO group and ISO + H_2_S group

The present study also confirmed that exposure to 0.75% isoflurane for 6 h could induce caspase-3 activation, as shown in Fig. 2, in both the hippocampus and cerebral cortex (both *p* < 0.01, vs CON group), but the H_2_S does not increased it. However, compared with the ISO group, the expression of cleaved caspase-3 decreased significantly in the H_2_S + ISO group, in both the hippocampus (*p* < 0.01) and cerebral cortex (*p* < 0.01), even the cleaved caspase-3 levels in cerebral cortex was still higher in ISO + H_2_S group than that in CON group (*p* < 0.05).

### H_2_S improved cognitive impairment caused by exposure to 0.75% isoflurane

**Fig. 3:**
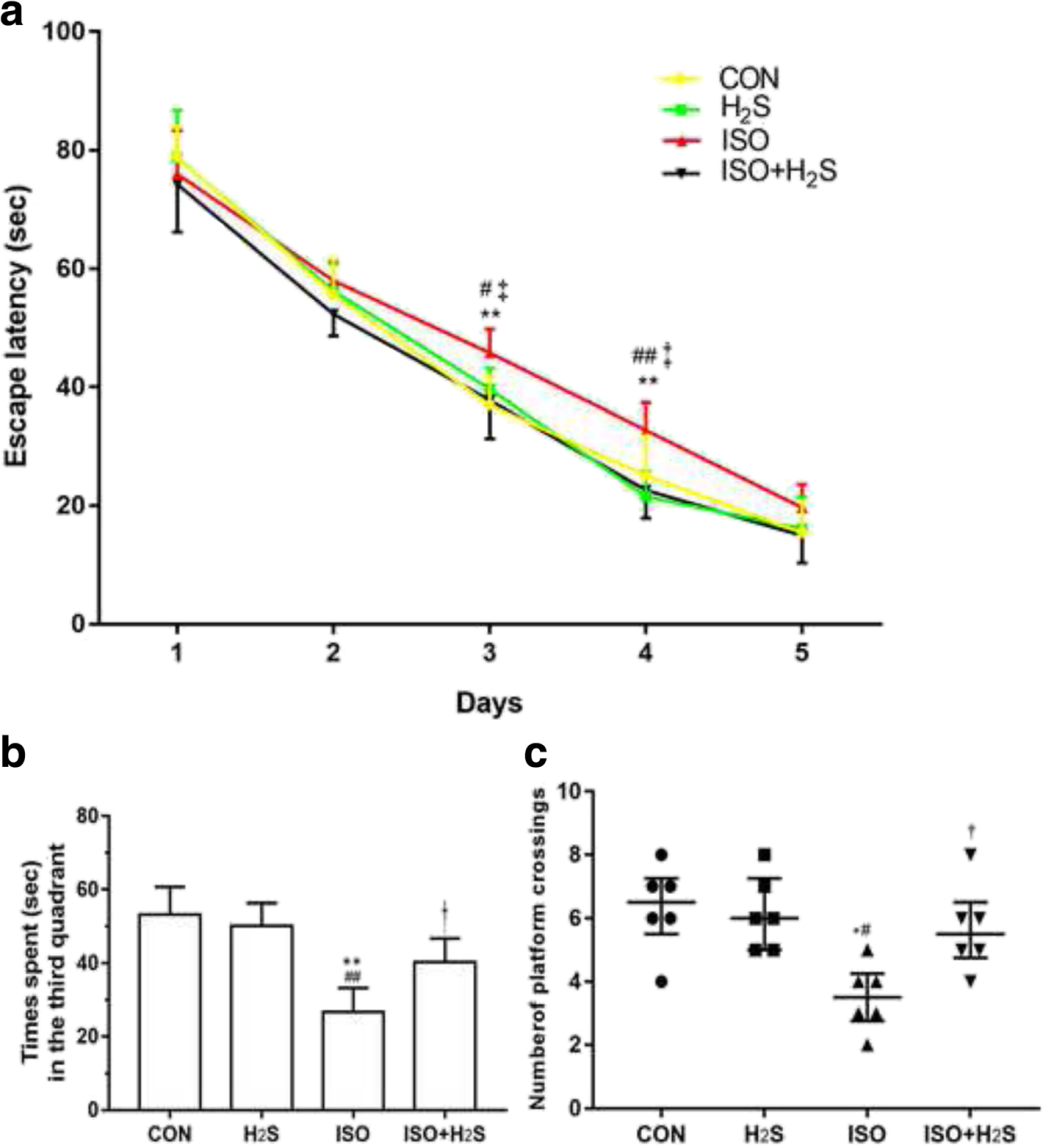
H2S attenuates isoflurane-induced cognitive impairments in the developing brain. Place trial demonstrating the latency time for the rats to arrive at the platform measuring spatial information acquisition (**a**). Probe trial demonstrating the time spent in the target (the third) quadrant (**b**) and the number of original platform crossings (**c**) measuring memory retention capabilities. Data represent as Mean ± SD (*n* = 6) in **a** and **b**, as median and interquartile range in **c**. * *p* < 0.05, ** *p* < 0.01 presented a significant difference compared with CON group; # *p* < 0.05, ## *p* < 0.01 presented a significant difference compared with H_2_S group, † *p* < 0.05, ‡ *p* < 0.01 presented a significant difference between ISO group and ISO + H_2_S group

The results from the escape latency trials to evaluate the abilities of the P42 rats for swimming and visual abilities in the MWM are summarized in Fig. 3a. Compared with the CON group, the average time to reach the submerged platform for the ISO group increased at P44, and P45 (*p* < 0.01, *p* < 0.01, respectively). And there was no different between CON group and H_2_S group during the training days. Compared with the H_2_S group, the average time to reach the submerged platform for the ISO group also increased at P44, and P45 (*p* < 0.05, *p* < 0.01, respectively). In addition, the significant differences was also found between ISO group and ISO + H_2_S group at P44 and P45 (*p* < 0.01, *p* < 0.01, respectively). In the probe test, the rats in the ISO group spent less time in the third quadrant where the platform was located (Fig. 3b) than that in the other groups (*p* < 0.01 vs the CON group, *p* < 0.01 vs the H_2_S group and *p* < 0.05 vs the ISO + H_2_S group). The number of crossings over the missing platform location were fewer in the ISO group (Fig. 3c) compared with the CON (*p* < 0.05), the H_2_S (*p* < 0.05) and the ISO + H_2_S group ((*p* < 0.05). Furthermore, there were no significant differences in swim speed during the probe trials (data not shown).

## Discussion

In the present study we demonstrated that exposure to 0.75% isoflurane for 6 h can significantly increase the level of activated caspase-3 in the hippocampus and cortex of P7 rats, and cause long-term cognitive impairment when they reached adulthood. However, administration of NaHS (a H_2_S donor) was found to decrease isoflurane-mediated neuroapoptosis and improve cognitive impairment.

Based on the British Journal of Anaesthesia, Salzburg Seminar, anesthesia neurotoxicity is influenced by many critical factors, such as the time and degree of the developing brain, the specific anesthetic drugs, the health status of the pups and specific procedure used for exposure to anesthetics [30]. The reason we chose the brain during the growth period is that it is susceptible to acute neural injuries, including by anesthetics. A previous study showed that P14 mice exposed to 1.7% isoflurane for 35 min daily for 4 successive days caused persistent, progressive memory dysfunction and reduced neurogenesis in young rodents, but not in P60 mice [31]. By contrast, H_2_S in the central nervous system is thought to have anti-apoptotic, anti-inflammatory and anti-oxidative benefits in mouse models of ischemia/hypoxia, Alzheimer’s disease and Parkinson’s disease [32]. Thus, we felt that it would be therapeutically meaningful to investigate whether H_2_S is beneficial against the neurotoxic effects of anesthetics such as neuroapoptosis and cognitive impairment.

Our data show that administration of NaHS decreased isoflurane-induced cleaved caspase-3 levels, which is a well-established biomarker of apoptosis, in the developing rat brain. These results suggest that the mechanism for H_2_S-induced neuroprotection occurs via activation of intrinsic or extrinsic anti-apoptotic pathways. However, this still needs further investigation. Fortunately, some previous findings seem to support our results. For example, it has been reported that endogenous H_2_S is a neuromodulator and it can mediate membrane ion channels, such as ATP-sensitive potassium (K_ATP_) channel and voltage-dependent calcium channels (VDCCs). The activation of K_ATP_ would hyperpolarization of neurons, reduce abnormal excitatory synaptic activity and are neuroprotective [33]. H_2_S can also indirectly inhibits L-type VDCCs currents in isolated mouse pancreatic β-cells [34] and rat cardiomyocytes [35], even another study in cerebellar granule neurons reported that H_2_S increased intracellular calcium ion concentration by stimulating L-type VDCCs [36]. Moreover, it can potentiate the effect of GABA and regulate stimulation of synaptic glutamate by astrocytes [37]. Interestingly, isoflurane can enhance and open GABA_A_ receptor channels directly [38] and open VDCCs. This increases intracellular calcium ion concentrations, which promote the activation of caspase-3 [11] or activate the mitochondrial apoptosis pathway [39]. Evidence demonstrate that H_2_S had anti-apoptotic effects via inhibition the forming and opening of mitochondrial permeability transition pores and the subsequent release of cytochrome C from mitochondria to the cytosol, as well as the activation of the caspase cascades [40]. H_2_S could protect neurons from oxidative stress via scavenging of reactive oxygen and/or nitrogen species (ROS and RNS) [41] and increasing production of glutathione which is a major and potent intracellular antioxidant [42]. H_2_S may also upregulate endogenous antioxidants through a nuclear-factor- E2-related factor-2 (Nrf2) dependent signaling pathway [43]. Evidence also finding H_2_S may stimulate glutamate transport function to protect brain from oxidative stress [44]. On the contrary, many anesthetics, even under normoxic conditions, can increase ROS, which cause neuronal lipid peroxidation and neuronal death in vulnerable brain regions such as the subiculum in the hippocampus [45]. H_2_S can also regulation intracellular signaling pathway to inhibit neuronal apoptosis. It can inhibits rotenone induced apoptosis via regulation of p38- and JNK-MAPK signaling pathway [40] and suppress H_2_O_2_-induced ERK 1/2 activation in primary cultured astrocytes [44]. H_2_S also acted via cAMP-mediated PI3K/Akt/p70S6K signal transduction pathways to inhibit hippocampal neuronal apoptosis and protect neurons from oxygen glucose deprivation/reoxygenation induced injury [46]. H_2_S also downregulates cytokines, such as tumor necrosis factor-α and interleukin-6 [47]. However, it has been found that clinically relevant isoflurane levels can increase the levels of tumor necrosis factor-α, interleukin-6, and interleukin-1β and thus may cause neuro-inflammation [48]. However, further research is needed to determine how H_2_S is neuroprotective. As mentioned before, H_2_S donors have shown beneficial therapeutic effects in neurodegenerative disease models. Some research also found that the brain levels of H_2_S in Alzheimer’s disease are lower than age-matched healthy controls [49], and that H_2_S attenuates deficits in cognition induced by ischemic stroke and surgical trauma [50, 51]. Therefore, H_2_S may be beneficial for extended use of anesthetics in maintaining long-term neurocognition. There are some reports on the causal link between anesthesia-induced neuroapoptosis and anesthesia-induced cognitive impairment. Studies have shown neuronal loss and perturbation of synaptic proteins are linked to cognitive ability [52], and neurogenesis is thought to play an important role in memory and learning in the hippocampus. Thus excessive apoptosis can affect the development of the central nervous system and even affect its physiological function [53]. In our study, the reduction of neuroapoptosis correlated with more pronounced memory impairment. The neural mechanisms of learning and memory depend not only on the structural plasticity of the central nervous system but also on the integrity of the neural network. Therefore, neuroapoptosis may affect the integrity of the neural network, thus impairing learning and memory [54, 55]. Our data suggest that reduced neuroapoptosis during brain development is important for cognitive development. However, Zhu et al. showed that P14 rats exposed to isoflurane for 4 consecutive days (35 min a day) did not have increased numbers of TUNEL-positive cells and active caspase-9 compared with the control group, or changes in synapsin I density, but did have impairment in reversal learning in rats [31]. These findings suggest some unknown mechanism of neurotoxicity by inhalation anesthetics, which cause cognitive deficits or cell death.

There were some limitations to our study. Although we found an effect of exogenous hydrogen sulfide on isoflurane-induced apoptosis and cognitive impairment in P7 rats, we did not delve into the mechanism of neuroprotection by H_2_S. However, several studies have demonstrated different signaling pathways of protection by H_2_S [56]. Therefore, in our next study we will explore the mechanism of neuroprotection by H_2_S, such as testing its effect on LTP on isoflurane-treated hippocampal slices.

## Conclusions

In conclusion, the present study shows that administration of H_2_S reduces neuroapoptosis and improves cognitive impairment caused by clinically relevant levels of isoflurane for 6 h in the developing rat brain. But further studies are needed to determine how H_2_S protects against anesthesia-induced apoptosis.

## Abbreviations

ANOVA: Analysis of variance
GABA: γ-aminobutyric acid
H_2_S: Hydrogen sulfide
K_ATP_: ATP-sensitive potassium channel
LTP: Long-term potentiation
MWM: Morris water maze
Nrf2: Nuclear-factor- E2-related factor-2
ROS/RNS: Reactive oxygen and/or nitrogen species
RT: Room temperature
SD: Standard deviation
TBST: Tris-buffered saline and Tween-20
TUNEL: Terminal deoxynucleotidyl transferase-mediated dUTP nick end-labeling
VDCCs: Voltage-dependent calcium channels
